# Comparative Analysis of Potential Risk Factors for at-Risk Gambling, Problem Gambling and Gambling Disorder among Current Gamblers—Results of the Austrian Representative Survey 2015

**DOI:** 10.3389/fpsyg.2017.02188

**Published:** 2017-12-14

**Authors:** Sven Buth, Friedrich M. Wurst, Natasha Thon, Harald Lahusen, Jens Kalke

**Affiliations:** ^1^Institute for Interdisciplinary Addiction and Drug Research (ISD), Association for Interdisciplinary Addiction and Drug Research (FISD e.V.), Hamburg, Germany; ^2^Centre for Interdisciplinary Addiction Research of Hamburg University, University Medical Centre Hamburg-Eppendorf, Hamburg, Germany; ^3^Psychiatric University Hospital Basel, Basel, Switzerland

**Keywords:** gambling, gambling disorder, risk factors, logistic regression, Austria

## Abstract

**Background:** The risk of developing a problem gambling behavior is distributed unequally among the population. For example, individuals who report stressful life events, show impairments of mental health or belong to a socio-economically deprived group are affected more frequently by gambling problems. The aim of our study is to investigate whether these risk factors are equally relevant for all gambling groups (social = 0 DSM-5 criteria, at risk = 1 DSM-5 criterion, problem = 2–3 DSM-5 criteria, disordered = 4–9 DSM-5 criteria).

**Methods:** Of a total of 10,000 participants in the representative gambling survey in Austria in 2015, 4,082 individuals reported gambling during the last 12 months and were allocated to the four gambling groups according to DSM-5. With social gamblers as the reference group, relevant risk factors for the other three groups were identified by means of bi- and multivariate multinomial logistic regression.

**Results:** Significant risk factors for gambling disorder are at-risk alcohol use (OR = 4.9), poor mental health (OR = 5.9), young age (≤26 years, OR = 2.1), a low level of formal education (OR = 2.4), having grown up with a single parent (OR = 2.5), parents with addiction problems (OR = 2.3) and belonging to the working class (OR = 2.9). Risk factors for problem gambling are parents with addiction problems (OR = 3.8), poor mental health (OR = 2.6) and a young age (OR = 2.2). With regard to at-risk gambling, only growing up with a single parent was relevant (OR = 2.4).

**Conclusion:** Overall, the results of this study suggest, that the number and the influence of the included risk factors differ between gambling problem groups. Apparently, the development of severe gambling problems is to a lesser extent facilitated by specific risk factors than by their cumulative presence. Therefore, future prevention and treatment measures should place a particular focus on individuals who have experienced growing up in a difficult family situation, have poor mental health, suffer from substance-related problems or have a low level of formal education.

## Introduction

In addition to genetic variables as a relevant factor for the development of problem gambling (Potenza et al., 2005; Black et al., 2014; Lobo et al., 2015), acceptance and availability of gambling, the cultural background of the person participating in gambling, the social and sociodemographic characteristics, as well as personality traits and mental health play an important role in the development of pathological gambling (Clarke, 2005).

With the exception of lotteries, gambling is a leisure activity performed more often by men than by women. Notably, men prefer types of gambling which are considered to be of particularly high risk for the development of problem gambling behavior, such as slot machines, casino games or sports betting (Hing et al., 2016). Therefore, it is not surprising that, compared to women, men are at a higher risk for developing gambling problems (Abbott et al., 2013; Barnes et al., 2015; Subramaniam et al., 2015). Age is another important demographic risk factor. Particularly younger age groups are disproportionately affected (Subramaniam et al., 2015; Abbott et al., 2016; Hing et al., 2016). Furthermore, individuals with a migration background develop a problem gambling behavior more frequently than persons who do not have such a background (Volberg et al., 2001; Buth, 2011; Hing et al., 2016).

Moreover, a low level of formal education (Fröberg et al., 2015; Subramaniam et al., 2015) and a social status below average (Volberg et al., 2001; Barnes et al., 2015) represent further relevant potential risk factors for problem gambling.

In addition to their gambling problems, many pathological gamblers are also affected by depressive or anxiety disorders (Barry et al., 2011; Bischof et al., 2013; Billi et al., 2014; Martin et al., 2014; Shultz et al., 2016). In a meta-analysis, Dowling et al. (2015) showed that an average of 75.5% of the pathological gamblers (currently in treatment) examined in the included studies, were affected by at least one additional comorbid mental disorder (axis I). More than half had been diagnosed with depression and about one quarter had an anxiety disorder. These mental health problems can be a cause as well as a consequence of problem gambling (Hodgins et al., 2005). However, regardless of causality issues, comorbid mental disorders indicate a higher risk of being affected by gambling problems.

Aside from the reported mental health impairments and certain personality traits, substance-related disorders are of great importance (Kessler et al., 2008; Barry et al., 2011; Bischof et al., 2013; Martin et al., 2014; Subramaniam et al., 2015; Shultz et al., 2016). In their meta-analysis Lorains et al. (2011) reported that 28.1% of pathological gamblers had an alcohol use disorder. With regard to illegal substances, the respective share was 17.2%. Similar to other addictive disorders, children of parents with a problem gambling behavior are at increased risk of developing gambling problems (Williams et al., 2015; Dowling et al., 2016). A number of studies on substance-related problems showed that having grown up with a single parent increased the risk of developing this sort of problem behavior (Blum et al., 2000; Latendresse et al., 2017). However, with regard to problem gambling, the effects of being raised by a single parent have been analyzed in only few studies. Ste-Marie (2005) found that the share of persons who grew up with single parents increased with the extent of the gambling problems. The studies by Canale et al. (2017) and Cheung (2014) also showed that persons who had not been raised by both parents had a higher risk of developing gambling problems.

The addictive potential of gambling varies with the different gambling products. While, in comparison, the use of lotteries and scratch cards leads to gambling problems rather rarely, sports betters, individuals who prefer casino games and especially persons who use slot machines are at higher risk of developing a gambling disorder (Scalese et al., 2016). This is particularly the case if the participation in these gambling forms occurs on a regular basis (Williams et al., 2015; Binde et al., 2017).

The above mentioned findings show that problem gambling is associated with a multitude of variables from various areas. Even though they do not always precede the development of gambling problems, these characteristics indicate a higher risk among affected individuals for also having a gambling problem. The results of the reported studies are predominantly outcomes of bivariate analyses, partly controlled by demographic variables. However, using these procedures, it cannot be excluded that the associations found are in fact the results of spurious relationships. The number and the importance of relevant factors of influence therefore might be overestimated. If however risk factors are simultaneously included in a multivariate analysis, the correlations between the variables included in the analysis are subtracted (controlled) and the effectively relevant factors can be determined.

Furthermore, the above mentioned studies are based on different definitions of problem gambling. While many studies only include individuals in the affected group who meet the criteria for pathological gambling (e.g., DSM-IV ≥ 5 criteria), other studies also include persons with problem (e.g., 3-4 DSM-IV criteria) or at-risk gambling behavior (1-2 DSM-IV- criteria). Although the latter procedure is understandable from a methodological perspective, as, particularly in representative surveys, the number of pathological gamblers is often too small for statistical analysis, it is nevertheless questionable when it comes to content. Thus, Shen et al. (2015) showed that moderate-risk gamblers (Problem Gambling Severity Index (PGSI): 3–7) differ significantly from problem gamblers (PGSI: ≥8) regarding psychological distress and possible alcohol dependence. Furthermore, the latter group participates significantly more frequently in poker games and sports betting and also takes part in online gambling considerably more often. However, given that both groups differ with regard to these characteristics, one may assume that factors which facilitate gambling problems are of varying importance within these groups.

Aim of the present study is to identify potential risk factors for disordered, problem, and at-risk gambling and to assess their respective relevance. If the analysis shows that the influence of variables varies with the severity of the gambling problem, existing treatment, protection and prevention measures would have to be adapted or new interventions would need to be developed from scratch for each of the individual problem groups.

## Methods

### Sample

The analysis is based on data of a general population survey on gambling behavior in Austria in 2015. The survey included sociodemographic and biographic data as well as data on gambling behavior, motives for gambling, alcohol use, mental health problems, suicidal thoughts and behavior as well as attitudes toward prevention measures (Kalke et al., 2016).

The basic population of the study consists of 14 to 65 year old inhabitants living in private households in Austria. This basic population is reduced to a sampling frame of German speaking individuals.

Data collection was conducted by means of computer assisted telephone interviews (CATI). The telephone numbers were drawn from public telephone directories (mobile and landline) using random sampling. The sample was stratified according to the number of inhabitants of each of the Austrian federal states.

Prior to the interview, the contacted individuals were asked to report the name of the federal state of their residence, their age and their gender. Only if the contacted person met the criteria of a yet not fully recruited quota, the full interview was carried out. In multiple-person households, the person with the next birthday coming up was interviewed (next-birthday-method). The interviews were conducted between January 9, 2015 and June 22, 2015.

A sample of 32,830 telephone numbers was drawn using the method described above. 11,890 numbers of this sample were neutral non-responses (invalid number, person not reached, no person in the target group e.g., regarding age, no private household, no communication possible). A total of 20,701 individuals had to be contacted in order to reach the targeted number of 10,000 interviews which equals a response rate of 48.3%. 18 cases were excluded from further analysis due to missing answers to DSM questions. Furthermore, only those 4,082 individuals who had reported gambling during the last 12 months were included in the following analyses.

Missing values were also found for other variables included in the analysis: professional status (*n* = 7); migration background (*n* = 18); parents with addiction problems (*n* = 37); growing up with a single parent (*n* = 10); alcohol problems (total score: *n* = 76); mental health problems (total score: *n* = 230). These missings were imputed with multiple imputation algorithms included in the statistical software MPlus 7.31 (Muthén and Muthén, 2015).

Despite the use of complex sampling procedures, achieving full representativeness of the sample is generally not possible. Therefore, the distributions in samples of representative surveys always differ slightly from those in the basic population. These differences are corrected post hoc by using weighting factors. The calculation of these weighting factors is based on the variables “federal state,” “age,” “gender,” and “formal education.” The weights were determined according to the distribution of these parameters among the Austrian general population.

This article is based on a secondary analysis of anonymized data from phone interviews for which all respondents gave oral consent before beginning the interview. They were free to withdraw at any time and without giving any reason. The data cannot be linked to the respondents. As the consultation of an ethics committee is not mandatory in the case of anonymized data collection and analysis, we refrained from requesting an ethics vote.

### Potential risk factors included in the analysis

The variables that should undergo testing were selected primarily on the basis of findings of other international studies which have investigated this issue (see above). As representative surveys are quite costly and respondents are only willing to participate in phone interviews for a limited amount of time, only items which could be validly assessed by means of very brief instruments such as Alcohol Use Disorder Identification Test-Consumption Questions (AUDIT-C) and the Mental Health Interview (MHI-5) were included. Apart from these two instruments, other risk factors included in the analysis were gender, age, highest qualification reached in school, migration background, addiction problems of parents, growing up with a single parent, professional status and participation in high risk gambling forms (sports betting, slot machines in and outside of casinos and casino games [i.e., roulette, poker]) on at least a monthly basis.

### Measures

#### Assessment of gambling problems

Gambling problems were operationalized using the criteria of the German language version of the Diagnostic and Statistical Manual of Mental Disorders (DSM-5) (Falkai et al., 2015). The DSM-5 provides nine criteria, which describe the main characteristics of a gambling disorder. These criteria were assessed using an instrument developed by Stinchfield (2002) for the DSM-IV which was adapted to DSM-5 by removing the criterion of having committed illegal acts to finance gambling. Stinchfield (2003) appraised the original instrument to be of satisfactory reliability, validity, and classification accuracy. The instrument adapted to DSM-5 includes 18 questions which can be answered with no (0) or yes (1). With the exception of criterion 4 (withdrawal symptoms), all criteria are operationalized through two individual questions. One criterion is met if at least one of the questions is answered with yes. If respondents meet four or more criteria, they are allocated to the group of gambling disorder. Respondents who meet two or three criteria are allocated to the group of problem gamblers. The group of at-risk gamblers meets only one of nine possible criteria.

As it can be assumed that individuals who only gamble occasionally or only spend small amounts of money on gambling do not develop gambling-related problems, the DSM-5 screening was conducted only for respondents who gambled at least once a week or spent at least 50 € per month.

#### Assessment of alcohol use

The 10-item AUDIT is a screening questionnaire developed by the WHO to identify harmful or hazardous alcohol consumption (Saunders et al., 1993). The AUDIT-C, consisting of the first three questions of the AUDIT (quantity, frequency and binge drinking) was developed as an even briefer, easy to administer screening measure. Both AUDIT and AUDIT-C are recommended by various guidelines. In this study, the AUDIT-C was used to assess alcohol use (Bush et al., 1998). The values of the predefined answers range from 0 to 4 points, with 12 points being the maximum total. The cut-off value in German speaking countries has been found to be 5 for men and women (Mann et al., 2016).

#### Assessment of mental health

The Mental Health Inventory-5 (MHI-5) was used as a screening instrument for mental health (Berwick et al., 1991). For Germany, the MHI-5 was validated by Rumpf et al. (2001) and was shown to be of satisfactory psychometric quality with regard to affective disorders and anxiety disorders.

The MHI-5 consists of five questions referring to nervousness, depressiveness with no possibility of solace, downheartedness and sadness, calmness, and happiness within the last 4 weeks. The five answer options range from “always” (1) to “never” (5). For the items calmness and happiness polarity needs to be reversed (recoding). Raw scores of 18 or less indicate problems in the area of mental health (Rumpf et al., 2001).

### Analysis

Common testing procedures were applied to test differences between the different gambling problem groups. These include the χ^2^-test (for dichotomous and categorical variables) as well as variance analysis procedures (for variables with a metrical measurement scale). In case of inhomogeneous variances of analyzed items, significance tests were conducted using the Welch-Test (Zimmerman, 2004).

The relevance of the included risk factors for the three problem groups (disorder, problem, at-risk) in comparison to the reference group (social gamblers) was initially tested by means of bivariate multinomial logistic regression analyses. For this procedure all four groups are included simultaneously in the analysis, but only one independent variable is included at a time (i.e., no controlling for third variables).

Bivariate analyses may allow for a first appraisal of the relevance of the included factors. However, this method cannot be used to assess whether the detected effects were possibly influenced by correlations with other potential risk factors. Therefore, in a second step, multivariate multinomial logistic regressions were conducted by simultaneously including all those potential risk factors in the analysis which were considered relevant and provided a sufficient number of cases for each problem gambling group. By doing so, correlations between different factors can be subtracted out (controlled).

In order to assess the strength of the association between the included factors, tetrachoric correlations were calculated on the basis of dichotomized items. The only exception was the DSM-5 which was included in the correlation analysis as a 4-step-scale (1 = “no criteria met,” 2 = “1 criterion met,” 3 = “2–3 criteria met,” and 4 = “4–9 criteria met”) (polychoric correlation).

Data preparation and calculation of χ^2^-tests, variance analyses and Welch-tests were performed with the use of the statistics program SPSS, version 15. The statistics software MPlus (Muthén and Muthén, 2015), version 7.31, was applied in order to perform the bivariate and multionomial regressions and to calculate the tetrachoric and polychoric correlations.

## Results

Of all respondents 40.9% (*N* = 4,082) reported to have participated in some kind of gambling within the last 12 months. The 12-month prevalence for a gambling disorder (DSM-5 ≥ 4 criteria) is 0.8% (*N* = 81). The number of respondents meeting 2 to 3 DSM-5 criteria is *N* = 72 (0.7%) and 121 (1.2%) meet one DSM-5 criterion (at-risk gambling). 3,808 respondents (38.8%) have participated in gambling within the last 12 months prior to the interview, but do not show any indications for gambling problems (social gambling).

In order to describe these four groups, a comparison was made regarding a range of variables which are considered as traditional risk factors for problem gambling (see introduction). The analysis shows that with growing severity of the gambling problems, the share of male gamblers increases (see Table 1). However, a significantly higher risk for males to be part of a problem group can only be found for disordered gamblers. Furthermore, individuals aged up to 26 bear a higher risk for disordered or problem gambling. Individuals with a migration background, a low level of formal education and the professional status of being a working class member are represented disproportionately strongly within the group of disordered gamblers. The corresponding odds ratios are only statistically relevant for this group.

**Table 1:** Potential risk factors for at-risk, problem, and disordered gambling – Results of the univariate logistic Regression.

		**Disordered (*****N*** = **81)**		**Problem (*****N*** = **72)**		**At-risk (*****N*** = **121)**		**Social (*N* = 3,808)**	**F/χ^2^**	***p*-value**
		**Mean/%**	**OR [95%-CI]^+^**	**Mean/%**	**OR [95%-CI] ^+^**	**Mean/%**	**OR [95%-CI] ^+^**	**Mean/%**		
Female gender [ref.]		21.0	3.2[1.7–6.1]	32.4%	1.8 [1.0–3.4]	34.9%	1.6 [1.1–2.4]	46.3%	χ^2^ = 31.6	*p* < 0.001
Age [ref. ≥27]	14–17	12.4%	3.6 [1.9–7.1]	8.5%	2.7 [1.3–5.6]	2.6%	1.0 [0.5–2.0]	1.3%	χ^2^ = 111.1	*p* < 0.001
	18–26	30.0%		26.8%		14.2%		15.4%		
	27–35	18.5%		27.8%		22.9%		18.6%		
	36–50	20.8%		19.2%		32.6%		34.5%		
	≥51	18.3%		17.7%		27.6%		30.1%		
	Mean (SD)	33.5 (13.2)		33.4 (12.5)		40.4 (12.5)		41.9 (13.3)	*F* = 19.8	*p* < 0.001
School-leaving qualification [ref: Intermediate secondary school or higher]	General secondary school	50.7%	4.9 [2.6–9.1]	27.5%	1.8 [0.9–3.8]	19.0%	1.1 [0.6–2.0]	17.4%	χ^2^ = 70.4	*p* < 0.001
	Intermediate secondary school	40.4%		53.4%		47.0%		51.8%		
	higher education entrance qualification/university degree	8.8%		19.1%		34.0%		30.8%		
Migration background [ref: no migration]		36.2%	3.4 [1.7–6.4]	17.1%	1.2 [0.6–2.4]	19.5%	1.4 [0.8–2.3]	14.7%	χ^2^ = 28.2	*p* < 0.001
At-risk alcohol use [ref: AUDIT-C ≤ 4]		63.8%	9.0 [4.8–16.9]	37.5%	3.1 [1.6–6.0]	28.1%	1.9 [1.2–3.1]	16.7%	χ^2^ = 136.5	*p* < 0.001
	Mean (SD)	5.3 (3.3)		3.7 (3.0)		3.1 (2.9)		2.6 (2.0)	*F* = 20.2	*p* < 0.001
Mental health problems [ref: MHI-5 ≥ 19]		71.0%	8.0 [3.6–17.6]	47.5%	3.1 [1.6–6.2]	24.4%	1.1 [0.7–1.8]	23.1%	χ^2^ = 102.2	*p* < 0.001
	Mean (SD)	15.9 (3.7)		18.8 (3.2)		20.0 (3.0)		20.1 (2.9)	*F* = 33.5	*p* < 0.001
Having grown up with a single parent [ref: with both parents]		43.0%	4.5 [2.4–8.4]	27.2%	2.2 [1.1–4.3]	30.0%	2.5 [1.6–4.0]	14.5%	χ^2^ = 76.7	*p* < 0.001
Parents with gambling- or substance-related problems [ref: parents without such problems]		46.0%	5.3 [2.8–9.8]	43.0%	4.6 [2.5–8.7]	17.7%	1.3 [0.8–2.2]	14.0%	χ^2^ = 106.0	*p* < 0.001
Professional status [ref. not working class]	Working class	43.6%	5.9 [3.1–11.1]	14.7%	1.3 [0.5–3.3]	17.0%	1.6 [0.8–2.9]	11.6%	χ^2^ = 106.6	*p* < 0.001
	Salaried employee/civil servant	11.1%		29.2%		34.2%		43.1%		
	Freelancer	14.9%		26.1%		18.8%		12.1%		
	Not employed	30.4%		30.0%		30.1%		33.2%		
Gambling forms (gambling at least monthly) [ref: respective participation less than monthly]	Sports Betting	60.4%	36.7 [19.2–70.1]	35.8%	13.4 [6.9–26.1]	22.6%	7.0 [4.2–11.8]	4%	χ^2^ = 605.9	*p* < 0.001
	Casino Games	11.8%	12.4 [4.5–34.0]	22.7%	27.1 [12.2–60.1]	15.5%	16.9 [8.1–35.3]	1.1%	χ^2^ = 305.1	*p* < 0.001
	Slot-Machines (Casino)	2.5%	14.1 [2.0–100.5]	3.3%	18.6 [2.9–119.8]	3.5%	19.7 [2.4–158.9]	0.2%	χ^2^ = 53.5	*p* < 0.001
	Slot-Machines (arcade halls, Bars, Restaurants)	31.0%	64.5 [26.2–158.8]	5.6%	8.6 [2.4–30.9]	5.6%	8.6 [2.9–25.4]	0.7%	χ^2^ = 506.7	*p* < 0.001

*+ = Reference category for the dependent variable for bivariate multinomial logistic regression: social gamblers; SD, standard deviation; OR, odds ratio; CI, confidence interval*.

The situation is different regarding the experience of growing up with a single parent. Although this constellation is most frequent among disordered gamblers, this item also constitutes a relevant risk factor among problem and at-risk gamblers.

More than 4 out of 10 disordered and problem gamblers further report to have parents with gambling- or substance-related problems of their own. For this group, the risk of being a disordered or problem gambler is increased by factor 5. In the group of at-risk gamblers however, the odds ratios do not differ significantly.

Almost two thirds of disordered gamblers show at least at-risk use of alcohol. With 37.5%, this share is considerably smaller among problem gamblers, but the percentage is still 20 points higher than among social gamblers. Also at-risk gamblers are disproportionately strongly affected by alcohol-related problems. Therefore, an at least at-risk use of alcohol is a relevant risk factor for all three problem groups.

A similar distribution was found regarding mental health. 71.0% of the disordered gamblers and almost half of the problem gamblers show psychological distress. However, among at-risk and social gamblers, this share amounts to less than a quarter. With odds ratios of 8.0 for disordered gambling and 3.1 for problem gambling, this item proves to be one the most relevant risk factors for gambling-related problems.

Table 1 further shows that at least monthly participation in sports betting, casino games and slot machines significantly increases the risk of being part of one of the three gambling problem groups. Within the group of disordered gamblers this is particularly true for sports betting and slot machines in gambling halls, bars and restaurants. On the other hand, only very few respondents reported such an intense gambling behavior. Therefore, the number of cases included in the analysis is small and correspondingly the confidence intervals of the OR are very wide. Thus, the logistical regression cannot provide reliable information on the importance of different gambling forms for the particular gambling problem groups. For this reason, the different forms of gambling are not included in the following multivariate analysis.

Multivariate analyses allow for controlling the effects of third variables. For this reason, a multivariate multinomial logistic regression was calculated for the factors presented in Table 2. The results show that at-risk alcohol use (OR = 4.0) and impaired mental health (OR = 5.9) are still particularly relevant risk factors for disordered gamblers. However, through controlling the influence of all other variables, the respective odds ratios turn out lower than in the bivariate analyses. This is also true for all other significant factors such as young age (OR = 2.1), low formal education (OR = 2.4), growing up with a single parent (OR = 2.5), having parents with addiction problems of their own (OR = 2.3), as well as being a working class member (OR = 2.9), whereas the factors of migration background and gender are no longer of statistically significant influence. Notably less statistically significant risk factors can be found for the group of problem gamblers. These are young age (OR = 2.2), impaired mental health (OR = 2.6), and having parents with gambling or substance-related problems (OR = 3.8). Remarkably, at-risk alcohol use is no longer of (statistical) relevance for the group of problem gamblers.

**Table 2:** Potential risk factors for disordered, problem, and at-risk gambling–Results of the multivariate multinomial logistic regression.

**Risk factors**	**Disordered (*****N*** = **81)**			**Problem (*****N*** = **72)**			**At-risk (*****N*** = **121)**		
	**OR^+^**	**95%-CI**	***p***	**OR^+^**	**95%-CI**	***p***	**OR^+^**	**95%-CI**	***p***
Male gender [ref. Female gender]	1.8	[0.9–3.8]	0.111	1.6	[0.8–3.3]	0.180	1.4	[0.9–2.1]	0.104
Age ≤ 26 years old [ref. ≥27]	2.1	[1.1–4.2]	0.036	2.2	[1.0–4.9]	0.045	0.8	[0.4–1.6]	0.512
Low formal education [ref: Intermediate secondary school or higher]	2.4	[1.2–5.0]	0.014	1.3	[0.6–2.8]	0.479	1.0	[0.6–1.8]	0.975
Migration background [ref: no migration]	1.8	[0.9–3.6]	0.123	0.9	[0.4–2.0]	0.768	1.3	[0.8–2.2]	0.355
At-risk alcohol use	4.0	[2.0–8.2]	0.000	1.9	[0.9–4.0]	0.101	1.6	[1.0–2.6]	0.052
Mental health problems [ref: MHI-5 ≥ 19]	5.9	[2.7–13.1]	0.000	2.6	[1.3–5.3]	0.006	1.1	[0.7–1.7]	0.836
Having grown up with a single parent [ref: with both parents]	2.5	[1.2–5.0]	0.011	1.5	[0.7–3.2]	0.337	2.4	[1.6–3.8]	0.000
Parents with addiction problems of their own [ref: parents without such problems]	2.3	[1.1–4.5]	0.019	3.8	[1.9–7.6]	0.000	1.1	[0.7–1.9]	0.596
working class [ref. no working class member]	2.9	[1.4–6.1]	0.003	0.8	[0.3–2.3]	0.718	1.3	[0.7–2.4]	0.482

*+ = Reference category for the dependent variables for multivariate multinomial logistic regression: social gamblers (0 DSM-IV-criteria; N = 3,808) OR, odds ratio; CI, confidence interval*.

With regard to at-risk gambling, having grown up with a single parent remains the only statistically significant risk factor. Among persons who report this, the risk of meeting exactly one DSM-5 criterion is increased by a factor of 2.4 compared to individuals who have grown up with both parents.

The results of the multivariate logistic regression are largely consistent with those of the bivariate analyses. Here, migration background and particularly gender constitute an exception as these two variables are no longer significant in the multivariate model. It is reasonable to assume that disproportionately high correlations with other included variables exist. In fact, the factor migration background correlates considerably with several of the included variables (see Table 3). This is the case for age of less than 27 years, having grown up with a single parent and low formal education. Even higher correlations could be found regarding gender. Here strong correlations exist with risky alcohol use (*r* = 0.55) and the professional status of being a working class member (*r* = 0.45). Another high correlation was identified with regard to age (*r* = 0.23). Furthermore, at-risk alcohol use correlates disproportionately highly with age (*r* = 0.30) and professional status (*r* = 0.34).

**Table 3:** Tetrachoric and polychoric correlations of relevant potential risk factors.

	**DSM-5**	**(I)**	**(II)**	**(III)**	**(IV)**	**(V)**	**(VI)**	**(VII)**	**(VIII)**
(I): Male gender	0.21^***^								
(II): Age ≤ 26 years old	0.22^***^	0.23^***^							
(III): Low formal education	0.24^***^	0.03	0.17^***^						
(IV): Migration background	0.18^***^	0.07^*^	0.15^***^	0.16^***^					
(V): At-risk alcohol use	0.38^***^	0.55^***^	0.30^***^	0.07^*^	0.07				
(VI) Mental health problems	0.32^***^	−0.11^***^	0.01	0.16^***^	0.09^**^	0.13^***^			
(VII): Having grown up with a single parent	0.31^***^	0.02	0.20^***^	0.19^***^	0.17^***^	0.10^**^	0.12^***^		
(VIII): Parents with addiction problems	0.33^***^	−0.09^**^	−0.03	0.15^***^	0.12^***^	0.13^***^	0.22^***^	0.26^***^	
working class	0.27^***^	0.45^***^	0.08^*^	0.14^***^	0.04	0.34^***^	0.07^*^	0.06	0.14^***^

Tetrachoric and polychoric correlations based on dichotomized risk factors; DSM-5, ordinal (4 categories) Significance:

*** p < 0.001;

** p < 0.01;

* *p < 0.05*.

## Discussion

The study at hand is the first internationally to assess risk factors for gambling-related problems as a function of problem severity according to DSM-5. With regard to disordered gamblers, the analysis showed that impaired mental health is an import risk factor. This finding is consistent with the results of various other studies (Kessler et al., 2008; Barry et al., 2011; Bischof et al., 2013; Martin et al., 2014; Subramaniam et al., 2015; Shultz et al., 2016). The same applies to at-risk alcohol use (Lorains et al., 2011; el-Guebaly et al., 2015; Williams et al., 2015). The level of correlation with the DSM-5 problem status is indeed above average for both impaired mental health and at-risk alcohol use, while inter-correlation between both variables is rather low (*r* = 0.13). Due to the cross-sectional design of the study, two different interpretations are possible here. One interpretation is that mental health problems and at-risk alcohol use are two different ways to react to gambling problems and their consequences. On the other hand, disordered gambling could be interpreted as a consequence of psychological and substance-related problems. In this case, both factors would constitute classical risk factors. However, due to the absence of a longitudinal design, this issue cannot be solved with the existing data. Nevertheless, individuals with impaired mental health and at-risk alcohol use constitute an important risk group for disordered gambling on which future prevention and therapy measures should place a greater focus.

For the remaining statistically relevant items, the direction of the correlation is not an issue. Either they cannot be influenced by gambling behavior (e.g., gender or age) or the development of these characteristics precedes the emergence of gambling problems. This is the case for young age (≤26 years old), low formal education, the professional status of being a working class member, having parents who have addiction problems or having grown up with a single parent. While the former have been confirmed as relevant factors by a multitude of studies, the influence of growing up with a single parent has yet hardly been analyzed. A significant odds ratio of 2.5 indicates that this group is particularly vulnerable for developing a disordered gambling behavior. A possible explanation is provided by Black et al. (2012). Here, worse family functioning in comparison to non-disordered gamblers was found to be an important risk factor for disordered gambling. As single parents are on their own regarding the organization of family life, it is fair to assume that problems accumulate in such cases. Furthermore, the precarious financial situation which single parents are often confronted with, is also associated with worse family functioning (Mansfield et al., 2013). Children who grow up with single parents also more often have at least one parent who is affected by gambling- or substance-related problems (*r* = 0.26). Therefore, the risk of transgenerational transmission of addiction is increased within this group (Vassoler et al., 2014).

It seems somewhat surprising that within the multivariate model, gender should not make a statistically significant difference between disordered gamblers and social gamblers. However, a close look at the correlations suggests that the alleged gender effect may rather be an effect of the social milieu. For at-risk use of alcohol, young age, and the status “working class” are associated closely. As such a milieu is dominated by males these variables also highly correlate with gender. If the reciprocal effects of these variables are controlled in a multivariate model, the influence of the gender variable is reduced considerably. This allows for the conclusion that the affiliation with a milieu of adolescent workers and drinkers significantly increases the risk for disordered gambling. Here, the gender of those affiliated with the milieu is not the decisive factor. Sharpe (2002) has identified the association between the drinker's milieu and gambling problems. She argues that slot machines are available in many bars and pubs where drinkers meet and that they are therefore frequently in contact with this highly addictive form of gambling.

Far fewer risk factors were identified for the group of problem gamblers. Among these are young age, mental health impairments, as well as parents with gambling- and substance-related problems. The disproportionately high correlation between the two latter variables (*r* = 0.22) can be interpreted as an indication that growing up with parents who have addiction problems of their own leads to mental health problems which are in turn suppressed by gambling (maladaptive coping). In many respects, this behavior would correspond to the development which Blaszczynski and Nower (2002) describe for the subgroup of emotionally vulnerable problem gamblers in their pathways model. If this is the case, problem gambling can be understood as a precursor for disordered gambling. In order to prevent this development in the future, there would be a need for prevention measures which convey the knowledge and skills to respond to existing mental health problems in another way.

At-risk gambling behavior cannot be predicted by means of traditional risk factors. Solely the factor of having grown up with a single parent was found to be statistically significant in the multivariate model. As this variable was also relevant for disordered gambling, special attention should be paid to children and youths growing up in such family constellations. The reasons why individuals who have undergone this kind of socialization develop a risk or disordered gambling behavior need to be investigated in further studies. Apart from the above mentioned dysfunctional family structures and parents' addiction problems, further important characteristics could be a lack of monitoring by parents, low problem-solving skills, low self-esteem and the company of other youths who are at risk of developing gambling problems themselves.

Bastiani et al. (2013) also tested the relevance of different risk factors for gambling problem groups (CPGI-classification: no risk, low-risk, moderate-risk or problem gambling) by means of a multivariate multinomial logistic regression. While gender and age were relevant for both problem groups, low to medium formal education and tobacco use we only relevant for moderate-risk or problem gamblers.

Furthermore, (Bischof et al., 2013) analyzed the importance of substance-related as well as anxiety and mood disorders for the affiliation with gambling problem groups, but could not find any differences between these groups. However, the study was based on a slightly different allocation of problem groups (DSM-IV, pathological: 5–10 criteria, problem: 3–4 criteria, risk: 1–2 criteria) and only clinically manifest disorders were included in the analysis, whereas the study at hand used brief screening instruments which also identify more moderate forms of these disorders.

Due to the small number of cases within the problem groups, the different gambling forms were not included in the multivariate analyses. Nevertheless, the univariate analyses confirm the relevance of a regular participation in sports betting, casino games and slot machines for the emergence of gambling-related problems (Williams et al., 2015; Binde et al., 2017). It is notable in this regard that the share of individuals who gamble in casinos is disproportionally high in the at-risk and problem gambling groups. Whereas many gamblers who use slot machines in gambling halls, bars and restaurants can be found in the group of disordered gamblers. The shares of sports betters are large in all gambling problem groups. However, in this context one needs to take into account that in Austria sports betting is performed more frequently on a regular basis than casino games or gambling at slot machines. As a whole, the results show that future prevention measures should focus particularly on these three and similar high-risk gambling forms.

Furthermore, it is a recognized fact that gamblers switch between different gambling problem groups throughout their gambling careers. Therefore, the gambling problem groups identified in this article only reflect the situation at the time of the interview.

## Limitations

Some limitations should be considered regarding the above mentioned results. Younger respondents, individuals from educationally deprived strata, and interviewees with a migration background were underrepresented in the given sample. Weighting was an attempt to ensure representativeness—at least with respect to the two first mentioned variables. Furthermore, interviews were only conducted with individuals who felt capable of being interviewed by phone and in German language. However, these limitations apply to almost all studies focusing on the general population—regardless of their particular topics. Moreover, the study at hand may slightly underestimate the number of individuals which meet the DSM-5 criteria, as it only included respondents who either play at least once a week or spend at least 50 € per month on gambling in the screening.

Finally, this is a cross-sectional study. This entails that, in strictly methodological terms, the relationships between at-risk/problem/disordered gambling and the other variables included in the analysis are only correlations. The assumptions which have been made particularly in the discussion section of this article are based on content-related considerations which would need to be tested in a longitudinal study.

## Conclusion

The results of this study suggest that the amount and the influence of statistically relevant risk factors differ among the three analyzed gambling problem groups. The differences between the respective gambling problem groups can hardly be explained with substantially different influence factors. Instead, the accumulation of existing risk factors seems to facilitate the development of gambling problems. Therefore, future prevention and treatment measures should focus especially on individuals who have grown up in difficult family situations, whose mental health is impaired, and who show substance-related disorders. Particularly this should be the case if they come from educationally deprived families. Apparently, adolescents with mental health or alcohol problems are at a particular risk of additionally developing gambling problems. This needs to be considered by facilities which provide counseling and treatment for the former problems, in order to prevent an additional development of gambling problems.

## Author contributions

JK, NT, FW, and SB: designed the study together and organized the data collection; SB: was responsible for the analyses and the draft of the manuscript; NT, FW, and JK: participated in the interpretation of the data and results; HL and FW: critically revised the manuscript for important intellectual content and approved the final version to be published.

### Conflict of interest statement

The research was supported by a grant from the Society for the research on Behavioral addictions, Austria [Gesellschaft zur Erforschung nicht stoffgebundener Abhängigkeiten” (GES)]. GES received financial support from Casinos Austria AG and Austrian Lotteries (Österreichische Lotterien GmbH).

## References

[B1] Abbott M. Stone C. A. Billi R. Yeung K. (2016). Gambling and problem gambling in Victoria, Australia: changes over 5 years. J. Gambl. Stud. 32, 47–78. 10.1007/s10899-015-9542-1 25895650

[B2] Abbott M. W. Romild U. Volberg R. A. (2013). Gambling and problem gambling in Sweden: changes between 1998 and 2009. J. Gambl. Stud. 30, 985–999. 10.1007/s10899-013-9396-3 23832754

[B3] Barnes G. M. Welte J. W. Tidwell M. C. Hoffman J. H. (2015). Gambling and substance use: co-occurrence among adults in a recent general population study in the united states. Int. Gambl. Stud. 15, 55–71. 10.1080/14459795.2014.990396 25914605 PMC4405260

[B4] Barry D. T. Stefanovics E. A. Desai R. A. Potenza M. N. (2011). Gambling problem severity and psychiatric disorders among Hispanic and white adults: findings from a nationally representative sample. J. Psychiatr. Res. 45, 404–411. 10.1016/j.jpsychires.2010.07.010 20800852 PMC3651857

[B5] Bastiani L. Gori M. Colasante E. Siciliano V. Capitanucci D. Jarre P.. (2013). Complex factors and behaviors in the gambling population of Italy. J. Gambl. Stud. 29, 1–13. 10.1007/s10899-011-9283-8 22138931

[B6] Berwick D. M. Murphy J. M. Goldman P. A. Ware J. E. Jr. Barsky A. J. Weinstein M. C. (1991). Performance of a five-item mental health screening test. Med. Care 29, 169–176. 10.1097/00005650-199102000-00008 1994148

[B7] Billi R. Stone C. A. Marden P. Yeung K. (2014). The Victorian Gambling Study: A Longitudinal Study of Gambling and Health in Victoria 2008-2012. Victoria, VIC: Victorian Responsible Gambling Foundation.

[B8] Binde P. Romild U. Volberg R. A. (2017). Forms of gambling, gambling involvement and problem gambling: evidence from a Swedish population survey. Int. Gambl. Stud. 17, 490–507. 10.1080/14459795.2017.1360928

[B9] Bischof A. Meyer C. Bischof G. Kastirke N. John U. Rumpf H.-J. (2013). Comorbid Axis I-disorders among subjects with pathological, problem, or at-risk gambling recruited from the general population in Germany: results of the PAGE study. Psychiatry Res. 210, 1065–1070. 10.1016/j.psychres.2013.07.026 23962739

[B10] Black D. W. Coryell W. H. Crowe R. R. McCormick B. Shaw M. C. Allen J. (2014). A direct, controlled, blind family study of DSM-IV pathological gambling. J. Clin. Psychiatry 75, 215–221. 10.4088/JCP.13m08566 24500179 PMC4221079

[B11] Black D. W. Shaw M. C. McCormick B. A. Allen J. (2012). Marital status, childhood maltreatment, and family dysfunction: a controlled study of pathological gambling. J. Clin. Psychiatry 73, 1293–1297. 10.4088/JCP.12m07800 23140646 PMC3514455

[B12] Blaszczynski A. Nower L. (2002). A pathways model of problem and pathological gambling. Addiction 97, 487–499. 10.1046/j.1360-0443.2002.00015.x 12033650

[B13] Blum R. W. Beuhring T. Shew M. L. Bearinger L. H. Sieving R. E. Resnick M. D. (2000). The effects of race/ethnicity, income, and family structure on adolescent risk behaviors. Am. J. Public Health 90, 1879–1884. 10.2105/AJPH.90.12.1879 11111260 PMC1446419

[B14] Bush K. Kivlahan D. R. McDonell M. B. Fihn S. D. Bradley K. A. (1998). The AUDIT alcohol consumption questions (AUDIT-C): an effective brief screening test for problem drinking. Ambulatory Care Quality Improvement Project (ACQUIP). Alcohol Use Disorders Identification Test. Arch. Intern. Med. 158, 1789–1795. 10.1001/archinte.158.16.1789 9738608

[B15] Buth S. (2011). Repräsentativbefragung der Bevölkerung, in Glücksspiel und Spielerschutz in Österreich: Empirische Erkenntnisse zum Spielverhalten der Bevölkerung und zur Prävention der Glücksspielsucht, eds Kalke J. Buth S. Rosenkranz M. Schütze C. Öchsler H. Verthein U. (Freiburg: Lambertus), 139–187.

[B16] Canale N. Vieno A. Griffiths M. D. Borraccino A. Lazzeri G. Charrier L.. (2017). A large-scale national study of gambling severity among immigrant and non-immigrant adolescents: the role of the family. Addict. Behav. 66, 125–131. 10.1016/j.addbeh.2016.11.020 27930902

[B17] Cheung N. W. (2014). Low self-control and co-occurrence of gambling with substance use and delinquency among Chinese adolescents. J. Gambl. Stud. 30, 105–124. 10.1007/s10899-012-9351-8 23224660

[B18] Clarke D. (2005). Factors leading to substance abuse, and implications for gambling in New Zealand. Int. J. Ment. Health Addict. 3, 41–52.

[B19] Dowling N. A. Cowlishaw S. Jackson A. C. Merkouris S. S. Francis K. L. Christensen D. R. (2015). Prevalence of psychiatric co-morbidity in treatment-seeking problem gamblers: a systematic review and meta-analysis. Aust. N. Z. J. Psychiatry 49, 519–539. 10.1177/0004867415575774 25735959 PMC4438101

[B20] Dowling N. A. Shandley K. Oldenhof E. Youssef G. J. Thomas S. A. Frydenberg E.. (2016). The intergenerational transmission of problem gambling: the mediating role of parental psychopathology. Addict. Behav. 59, 12–17. 10.1016/j.addbeh.2016.03.002 26999631

[B21] el-Guebaly N. Casey D. Shawn R. Hodgins D. C. Schopflocher D. Smith G. (2015). The Leisure, Lifestyle, & Lifecycle Project (LLLP): A Longitudinal Study of Gambling in Alberta. Alberta: Alberta Gambling Research Institute.

[B22] Falkai P. Wittchen H.-U. Döpfner M. American Psychiatric Association (2015). Diagnostisches und Statistisches Manual Psychischer Störungen DSM-5®. Göttingen: Hogrefe.

[B23] Fröberg F. Modin B. Rosendahl I. K. Tengström A. Hallqvist J. (2015). The association between compulsory school achievement and problem gambling among Swedish young people. J. Adolesc. Health 56, 420–428. 10.1016/j.jadohealth.2014.12.007 25659200

[B24] Hing N. Russell A. Tolchard B. Nower L. (2016). Risk factors for gambling problems: an analysis by gender. J. Gambl. Stud. 32, 511–534. 10.1007/s10899-015-9548-8 25948418 PMC4875054

[B25] Hodgins D. C. Peden N. Cassidy E. (2005). The association between comorbidity and outcome in pathological gambling: a prospective follow-up of recent quitters. J. Gambl. Stud. 21, 255–271. 10.1007/s10899-005-3099-3 16134008

[B26] Kalke J. Buth S. Thon N. Wurst F. M. (2016). Glücksspielverhalten der österreichischen Bevölkerung und ihre Akzeptanz von Spielerschutzmaßnahmen – Ergebnisse der Repräsentativbefragungen 2009 und 2015. Suchttherapie (eFirst). 10.1055/s-0042-121996

[B27] Kessler R. C. Hwang I. LaBrie R. Petukhova M. Sampson N. A. Winters K. C.. (2008). DSM-IV pathological gambling in the national comorbidity survey replication. Psychol. Med. 38, 1351–1360. 10.1017/S0033291708002900 18257941 PMC2293303

[B28] Latendresse S. J. Ye F. Chung T. Hipwell A. Sartor C. E. (2017). Parental monitoring and alcohol use across adolescence in black and white girls: a cross-lagged panel mixture model. Alcohol. Clin. Exp. Res. 41, 1144–1153. 10.1111/acer.13386 28391598 PMC5482355

[B29] Lobo D. S. S. Aleksandrova L. Knight J. Casey D. M. el-Guebaly N. Nobrega J. N.. (2015). Addiction-related genes in gambling disorders: new insights from parallel human and pre-clinical models. Mol. Psychiatry 20, 1002–1010. 10.1038/mp.2014.113 25266122

[B30] Lorains F. K. Cowlishaw S. Thomas S. A. (2011). Prevalence of comorbid disorders in problem and pathological gambling: systematic review and meta-analysis of population surveys. Addiction 106, 490–498. 10.1111/j.1360-0443.2010.03300.x 21210880

[B31] Mann K. Hoch E. Batra A. (2016). S3-Leitlinie Screening, Diagnose und Behandlung alkoholbezogener Störungen. Berlin; Heidelberg: Springer.

[B32] Mansfield A. K. Dealy J. A. Keitner G. I. (2013). Family functioning and income. Family J. 21, 297–305. 10.1177/1066480713476836

[B33] Martin R. J. Usdan S. Cremeens J. Vail-Smith K. (2014). Disordered gambling and co-morbidity of psychiatric disorders among college students: an examination of problem drinking, anxiety and depression. J. Gambl. Stud. 30, 321–333. 10.1007/s10899-013-9367-8 23430449

[B34] Muthén L. K. Muthén B. O. (2015). Mplus User's Guide. Los Angeles, CA: Muthén & Muthén.

[B35] Potenza M. N. Xian H. Shah K. Scherrer J. F. Eisen S. A. (2005). Shared genetic contributions to pathological gambling and major depression in men. Arch. Gen. Psychiatry 62, 1015–1021. 10.1001/archpsyc.62.9.1015 16143733

[B36] Rumpf H. J. Meyer C. Hapke U. John U. (2001). Screening for mental health: validity of the MHI-5 using DSM-IV Axis I psychiatric disorders as gold standard. Psychiatry Res. 105, 243–253. 10.1016/S0165-1781(01)00329-8 11814543

[B37] Saunders J. B. Aasland O. G. Babor T. F. de la Fuente J. R. Grant M. (1993). Development of the Alcohol Use Disorders Identification Test (AUDIT): WHO collaborative project on early detection of persons with harmful alcohol consumption–II. Addiction 88, 791–804. 10.1111/j.1360-0443.1993.tb02093.x 8329970

[B38] Scalese M. Bastiani L. Salvadori S. Gori M. Lewis I. Jarre P.. (2016). Association of problem gambling with type of gambling among italian general population. J. Gambl. Stud. 32, 1017–1026. 10.1007/s10899-015-9579-1 26475172

[B39] Sharpe L. (2002). A reformulated cognitive–behavioral model of problem gambling: a biopsychosocial perspective. Clin. Psychol. Rev. 22, 1–25. 10.1016/S0272-7358(00)00087-8 11793575

[B40] Shen Y. Kairouz S. Nadeau L. Robillard C. (2015). Comparing problem gamblers with moderate-risk gamblers in a sample of university students. J. Behav. Addict. 4, 53–59. 10.1556/2006.4.2015.002 26014673 PMC4500885

[B41] Shultz S. K. Shaw M. McCormick B. Allen J. Black D. W. (2016). Intergenerational childhood maltreatment in persons with DSM-IV pathological gambling and their first-degree relatives. J. Gambl. Stud. 32, 877–887. 10.1007/s10899-015-9588-0 26749583 PMC4939131

[B42] Ste-Marie C. (2005). Parenting Styles and Family Environment: Influences on Youth Problem Gambling. Montreal, QC: McGill University.

[B43] Stinchfield R. (2002). Reliability, validity, and classification accuracy of the South Oaks Gambling Screen (SOGS). Addict. Behav. 27, 1–19. 10.1016/S0306-4603(00)00158-1 11800216

[B44] Stinchfield R. (2003). Reliability, validity, and classification accuracy of a measure of DSM-IV diagnostic criteria for pathological gambling. Am. J. Psychiatry 160, 180–182. 10.1176/appi.ajp.160.1.180 12505822

[B45] Subramaniam M. Abdin E. Vaingankar J. A. Wong K. E. Chong S. A. (2015). Comorbid physical and mental illnesses among pathological gamblers: results from a population based study in Singapore. Psychiatry Res. 227, 198–205. 10.1016/j.psychres.2015.03.033 25912429

[B46] Vassoler F. M. Byrnes E. M. Pierce R. C. (2014). The impact of exposure to addictive drugs on future generations: physiological and behavioral effects. Neuropharmacology 76(pt B), 269–275. 10.1016/j.neuropharm.2013.06.016 23810828 PMC3864776

[B47] Volberg R. A. Abbott M. W. Ronnberg S. Munck I. M. (2001). Prevalence and risks of pathological gambling in Sweden. Acta Psychiatr. Scand. 104, 250–256. 10.1034/j.1600-0447.2001.00336.x 11722299

[B48] Williams R. Hann R. G. Schopflocher D. West B. McLaughlin P. White N. (2015). Quinte Longitudinal Study of Gambling and Problem Gambling. Guelph, ON: Ontario Problem Gambling Research Centre.

[B49] Zimmerman D. W. (2004). A note on preliminary tests of equality of variances. Br. J. Math. Stat. Psychol. 57(Pt 1), 173–181. 10.1348/000711004849222 15171807

